# 1005. COVID-19 At-Home Capillary Blood Specimen Collection Pilot – Gaining Insight into Independent Self- Collection of Blood Specimens and COVID-19 Within the Veteran Population

**DOI:** 10.1093/ofid/ofab466.1199

**Published:** 2021-12-04

**Authors:** Tseli Mohammed, Jessica V Brewer, Mary E Pyatt, Juan P Romero Casas, Stacey Whitbourne, J Michael Gaziano, Connor W Edson, Mark Holodniy

**Affiliations:** 1 VA Boston Healthcare System, Boston, Massachusetts; 2 Department of Veterans Affairs, Palo Alto, California

## Abstract

**Background:**

The VA Million Veteran Program (MVP) studies what factors influence Veteran health. Current procedures involve collection of venous blood at MVP enrollment sites. To examine home specimen collection options, MVP performed a pilot study comparing two blood specimen collection devices and evaluated SARS-CoV-2 antibody assays to determine known COVID-19 infection or vaccination.

**Methods:**

A sub-sample of MVP Veteran participants were asked to self-collect a capillary blood specimen using the Neoteryx Mitra Clamshell (up to 120uL dried blood) or Tasso-SST (up to 200uL liquid blood) per the vendor instructions. Veterans were randomly assigned to a device prior to consent. Eligibility included 30% of Veterans with known COVID-19 diagnosis or vaccination and sampling time was variable from these events. Veterans rated their device experience and shipped collected specimens directly to an MVP laboratory. Mitra tip (4) blood was eluted in 1 mL of 0.9% normal saline for 1 hour at room temperature shaking at 300 rpm. Tasso tubes were centrifuged per vendor instructions. All samples were stored at -80°C until tested with SARS-Cov-2 antibody (Ab) assays (InBios Spike IgG, BioRad Nucleocapsid (NC) Total Ab, Abbott NC IgG, and Abbott Spike IgG II) per vendor instructions.

**Results:**

312 MVP participants consented to the pilot (52%) of which 136 (43.6%) were sent Mitra and 176 (56.4%) were sent Tasso-SST (Table 1). Participants rated the Mitra Tasso-SST equally on average as 4.4 on a 0-5 usability scale. The Abbott IgG II assay had the highest sensitivity across both devices (87% Mitra and 98% Tasso-SST) for detecting known COVID infection and/or vaccination. The InBios IgG assay with the Tasso-SST had the best sensitivity (97%) and specificity (80%) for detecting known COVID-19 infection and/or vaccination (Table 2).

Table 1. COVID-19 At-Home Capillary Blood Specimen Collection Pilot Outcomes

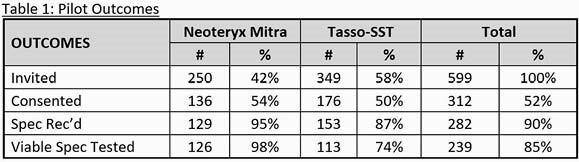

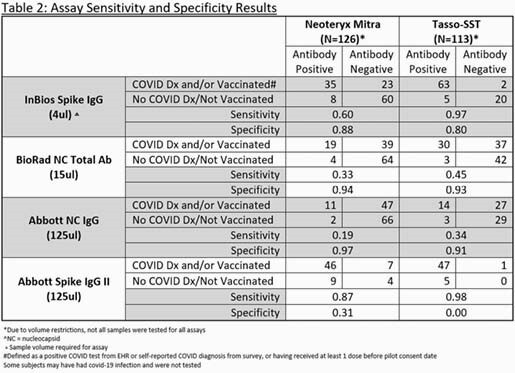

**Conclusion:**

Veterans successfully collected their own specimens and had no strong preference for either device. The Tasso-SST combined with the InBios Spike IgG assay provided the highest combination of sensitivity and specificity. Limitations included one collection device per subject, varied timing of testing, unknown infection or vaccination status among some, and Tasso collection volume and Mitra whole blood dilution may have affected comparison across assays or performance.

**Disclosures:**

**All Authors**: No reported disclosures

